# Exploring Flip Flop memories and beyond: training Recurrent Neural Networks with key insights

**DOI:** 10.3389/fnsys.2024.1269190

**Published:** 2024-03-27

**Authors:** Cecilia Jarne

**Affiliations:** ^1^Departamento de Ciencia y Tecnologia de la Universidad Nacional de Quilmes, Bernal, Quilmes, Buenos Aires, Argentina; ^2^CONICET, Buenos Aires, Argentina; ^3^Department of Clinical Medicine, Center of Functionally Integrative Neuroscience, Aarhus University, Aarhus, Denmark

**Keywords:** recurrent neural networks, dynamics, computational neuroscience, flip flop, eigenvalue distribution

## Abstract

Training neural networks to perform different tasks is relevant across various disciplines. In particular, Recurrent Neural Networks (RNNs) are of great interest in Computational Neuroscience. Open-source frameworks dedicated to Machine Learning, such as Tensorflow and Keras have produced significant changes in the development of technologies that we currently use. This work contributes by comprehensively investigating and describing the application of RNNs for temporal processing through a study of a 3-bit Flip Flop memory implementation. We delve into the entire modeling process, encompassing equations, task parametrization, and software development. The obtained networks are meticulously analyzed to elucidate dynamics, aided by an array of visualization and analysis tools. Moreover, the provided code is versatile enough to facilitate the modeling of diverse tasks and systems. Furthermore, we present how memory states can be efficiently stored in the vertices of a cube in the dimensionally reduced space, supplementing previous results with a distinct approach.

## 1 Introduction

Machine learning methods, especially Deep Learning, have achieved remarkable success across diverse tasks in various domains. These include speech processing (Ogunfunmi et al., 2019), bioinformatics (Min et al., 2016), where algorithms predict protein structures, discover drugs and analyze gene expression data, and image recognition (Litjens et al., 2017) where deep learning classifies images and detects objects.

The emergence of open-source frameworks dedicated to Machine Learning, such as Pytorch, Tensorflow and Keras (Abadi et al., 2015; Chollet et al., 2015; Paszke et al., 2019) has produced huge changes in the development of technologies we use every day for different tasks in research. Due to their novelty and complexity, it can be challenging to properly learn how to utilize these frameworks in different relevant scientific domains, such as the development of models in Computational Neuroscience, which will be the aim of the present work.

To bridge the gap between theoretical knowledge and practical application, clear tutorials or primers are crucial. These resources should equip researchers not only with the ability to implement the algorithms but also with the skills to solve diverse problems pertinent to their field.

Recurrent Neural Networks (or RNNs) were originally invented by Paul Werbos, who also invented backpropagation, a fundamental tool for training these models (Werbos, 1990). This also includes the concept of latent variables. The problem of training neural networks to perform different tasks is relevant across various disciplines that go beyond Machine Learning. In particular, RNNs are of great interest in different scientific communities. These models also have great relevance concerning control systems and other areas such as electronics (Alianna J. Maren and, Auth.; Deng, 2013; Dinh et al., 2014; Mohajerin and Waslander, 2017). One relevant problem to address with them is how to build models for the study of dynamical systems and how to extract meaningful information from them.

In general, Neural Networks are algorithms that allow us to model different systems. According to the Universal Approximation Theorem, a neural network with one hidden layer containing a sufficient but finite number of neurons can approximate any continuous function to a reasonable accuracy under certain conditions for activation functions (Hornik, 1991). This theorem has been extended to RNNs. It is well known that dynamical systems can be approximated by continuous-time RNNs (Funahashi and Nakamura, 1993).

In particular, RNNs are widely used in the field of Computational Neurosciences to describe the behavior of cortical areas, which presents great recurrence in their connections (Murphy and Miller, 2009). They are related to the processing of temporal information and the production of time-dependent outputs.

The basic premise of RNNs is that the feedforward connection weights in a Multilayer Perceptron (MLP) neural network (McCulloch and Pitts, 1943) can be modified using prior activation history as well as the immediately presented stimulus. This mechanism can be considered to encapsulate, in a very simple model, the much broader and more interesting task of guiding neural behavior. Factors that influence neural interactions and even growth can be included within this simple model. In this context, the broader scope of systems neuroscience relates to a detailed and careful analysis of RNNs.

The realm of temporal influence within systems neuroscience has a long and substantive history. The work by Levi-Montalcini and Booker (1960); Levi-Montalcini (1987), was among the earliest to show how specific signaling proteins (nerve growth factors, or NGFs) could influence temporal evolution within an organism. More recently, Baldassarro et al. (2023) showed, in an in vitro study, that NGFs could influence the proliferation of fetal brain multipotent stem cells, pushing them into a specific oligodendrocyte cell lineage and also influencing the differentiation of oligodendrocyte precursor cells. These works are simply examples of how the complex process of influencing neural cell growth and differentiation can be influenced over time, by introducing specific signaling mechanisms. For this, the notion of RNNs encapsulates a much larger suite of neural processes.

In this way, RNNs allow the incorporation of realistic characteristics at the biological level, such as Dale's law (Dale, 1935; Rajan and Abbott, 2006; Song et al., 2016; Jarne and Caruso, 2023), sparsity or different characteristics of interest in animal models.

In the field of Machine learning, more sophisticated architectures such as LSTM (Long Short Term Memory units) or GRU (Gated recurrent units) are widely spread and have been used to process temporal sequences since they do not have the same limitations as RNNs to process long time dependencies (Bengio et al., 1994; Pascanu et al., 2013; Chung et al., 2014; SHI et al., 2015; Gudowska-Nowak et al., 2020). Other powerful models are based on spiking neural networks (SNNs). Several recent studies have made significant contributions to the field of brain-inspired intelligence. These studies demonstrate the potential of this field to achieve high-level intelligence, high accuracy, high robustness, and low power consumption (Yang et al., 2022a,b, 2023; Yang and Chen, 2023a,b).

The primary reason for using simple RNN models lies in their ability to comprehend neural computation through collective dynamics, a phenomenon intricately linked to motor control, brain temporal tasks, decision-making (Mante et al., 2013), neural oscillations and working memory (Vyas et al., 2020; Jarne and Caruso, 2023; Pals et al., 2024).

Analyzing the dynamics inherent in these models allows us to formulate various hypotheses regarding the functioning of different brain areas and to offer an interpretation for the experimental results observed (Barak, 2017; Kao and Hennequin, 2019). An illustrative instance involves the recent utilization of RNNs to transfer learned dynamics and constraints to a spiking recurrent neural network in a one-to-one fashion (Kim et al., 2019).

A well-established fact is that the dynamics of a network are heavily influenced by the eigenvalue spectrum of the weight matrix describing synaptic connections (Zhou et al., 2009). Thus, the significance of investigating this distribution lies in elucidating various aspects of the dynamic behavior of the system, which is why, in Section 5.2, such analysis will be presented and described.

There are general tutorials available on artificial neural networks, such as Yang and Wang (2020). However, in this work, we will focus extensively on RNNs and their application in Computational Neuroscience because they play a relevant role in understanding complex neural processes and dynamics. Throughout this tutorial, we will delve into the architecture, training methodologies, and practical aspects of the RNN implementation. We explore also their significance and potential contributions to the field.

A simple RNN was chosen and trained to perform a time-series processing task inspired by Computational Neuroscience studies (Sussillo, 2014). The implementation of the network, the training, and the tools are carefully described here, as well as different forms to obtain the information that allows a suitable description of the system under study.

Training an RNN to perform temporal tasks has many difficulties and can be done through various paradigms. Here it is proposed to approach the problem through supervised learning. The entire procedure is described in detail.

**Figure 1:**
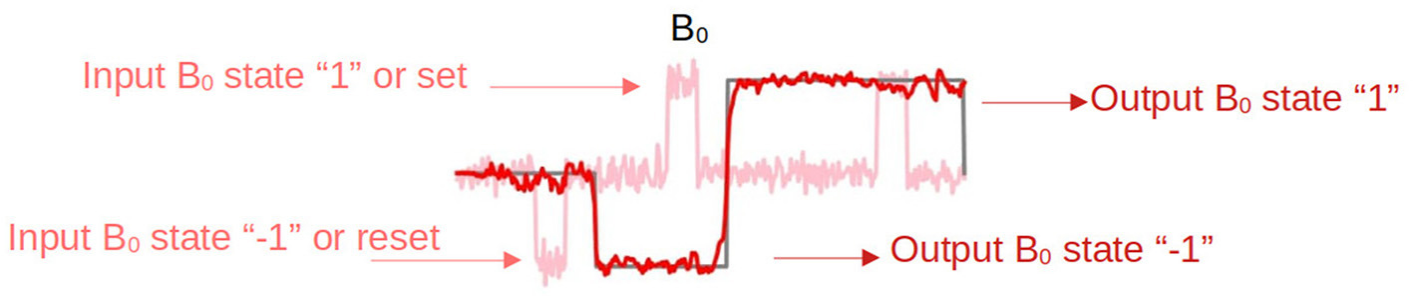
A Flip Flop. Binary task designed to store one bit of information. It has two inputs, Set (S) and Reset (R), which in here will be represented by the temporal signal states of *B*_0_ +1 and -1 in amplitude, and one output Q. The output represents the current state of the Flip Flop and can be either 1 or -1.

Among the different tasks, the Flip Flop was chosen as a case example. On one hand, a Flip Flop is the simplest sequential system that one can build Floyd (2003). To be precise, a 3-bit memory was studied, which is a task composed of a set of Flip Flops as the one shown in Figure 1. This is also a working memory task considered previously in other works in Computational Neuroscience (Sussillo and Barak, 2013; Barak, 2017; Jarne, 2022). The parameterization of the chosen task, one fundamental key in any work related to trained RNNs, is as described in Sussillo and Barak (2013). It is also revisited here. Gradient descendant minimization was used to take advantage of different optimized implementations of the available algorithms. The code implementation is presented using Tensorflow and Keras. The reason for this choice is that such scientific libraries are open-source, their use is rapidly growing, and they are becoming increasingly popular. One can find excellent documentation for software development about them Gulli and Pal (2017); Ramsundar and Zadeh (2018); Singh and Manure (2019). Also, we have new tools such as Google Colaboratory that allow implementing and testing models directly online.

The focus of this paper is on elucidating how a trained RNN operates, with code provided for detailed study. The “Flip Flop problem” is chosen to illustrate the study. Every step is thoroughly explained, from parameterizing the task to describing the dynamics of trained networks.This example is used to show how the problem of training networks can be studied using these computing tools applied in any temporal task in general, but also to discuss the limitations that networks have and the alternatives to solve them.

The rest of the paper is organized as follows. In Section 2, the description of the dynamics, discretization and code examples are presented. In Section 3, the task parametrization is shown. Section 4 describes the training protocol. In Section 5, the results, different analyses of the network, tools and software are discussed in detail. Finally, Section 6 includes the final remarks.

## 2 Model

The dynamics of the units in the RNN model is inspired by Equation 1, based on a model for a large network of neurons with a graded response (Hopfield, 1984).


(1) $$\begin{array}{l} \frac{dh_{i}\left(t\right)}{dt}=-\frac{h_{i}\left(t\right)}{\tau }+\sigma \left(\overset{N}{\underset{j=1}{\sum }}W_{ij}^{rec}h_{j}\left(t\right)+\overset{M}{\underset{k=1}{\sum }}W_{ik}^{in}x_{k}\left(t\right)\right) \end{array}$$

The dynamics of the RNN model of *N* units is described in terms of the activity column vector function $h={\left(h_{1},\cdots \;,h_{N}\right)}^{𝔱}$, where 𝔱 represent the matrix transposition. The *i*−activity component *h*_*i*_, where *i* = 1, ⋯ , *N* satisfies the differential equation as a function of time *t*. τ represent a characteristic time of the system and σ is a non-linear activation function. The elements $W_{ij}^{rec}$ are the synaptic connection strengths of to the recurrent weight matrix ***W***^*rec*^∈ℝ^*N*×*N*^ and ***x***_*k*_ are the component of the column vector function of input signal $x={\left(x_{1},\cdots \;,x_{M}\right)}^{𝔱}$. The elements $W_{ik}^{in}$ conform the input weight matrix ***W***^*in*^∈ℝ^*N*×*M*^ which connects the input signal ***x*** to each of *N* units with activity vector ***h***.

The network is fully connected, and matrices have weights given by a certain parametrization of interest. For the example, we considered a normal distribution with zero mean and variance $\frac{1}{N}$.

The network has three layers: the input, the recurrent hidden layer, and the output layer. The readout, in terms of the matrix elements $W_{i}^{out}$, from *W*^*out*^ is described by Equation 2.


(2) $$\begin{array}{l} z\left(t\right)=\overset{N}{\underset{i=1}{\sum }}W_{i}^{out}h_{i}\left(t\right). \end{array}$$

In terms of the output weight matrix, which in this work is a row vector, it could be written as:


(3) $$\begin{array}{l} W^{out}=\left(W_{1}^{out},\cdots \;,W_{N}^{out}\right). \end{array}$$

We considered σ() = *tanh*() and τ = 1, without loss of generality. The model is discretized using Euler's method following Ingrosso and Abbott (2019); Bondanelli and Ostojic (2020); Bi and Zhou (2020); Jarne (2022); Jarne and Laje (2023).

In vector form, the Equations 1 and 2 can be written as:


(4) $$\begin{array}{l} \frac{d\text{H(t)}}{dt}=-\frac{\text{H}\left(t\right)}{\tau }+\sigma \left({\text{W}}^{\text{Rec}}\text{H}\left(t\right)+{\text{W}}^{\text{in}}\text{X}\left(t\right)\right) \end{array}$$

and respectively:


(5) $$\begin{array}{l} \text{Z(t)}={\text{W}}^{\text{out}}\text{H}\left(t\right) \end{array}$$

The system represented by Equation 1 is approximated using Euler's method as previously indicated, with a step time δ*t*. A value of τ = 1 was considered. Then, the dynamics of the discrete-time RNN is given by Equation 6


(6) $$\begin{array}{l} \text{H}\left(t+\delta t\right)=\text{H}\left(t\right)+\left(-\text{H}\left(t\right)+\sigma \left({\text{W}}^{\text{Rec}}\text{H}\left(t\right)+{\text{W}}^{\text{in}}\text{X}\left(t\right)\right)\right), \end{array}$$

The value considered for the time step is δ*t* = 1 to obtain the time evolution. Usually, the amplitude of the activity *H*(*t*) is adimensional or expressed in arbitrary units. It will depend on context. Then, from Equation 6, the activity of the recurrent units at the next time step is given by Equation 7.


(7) $$H(t+1)=\sigma (W^{Rec}H(t)+W^{in}X(t)))$$

**Figure 2:**
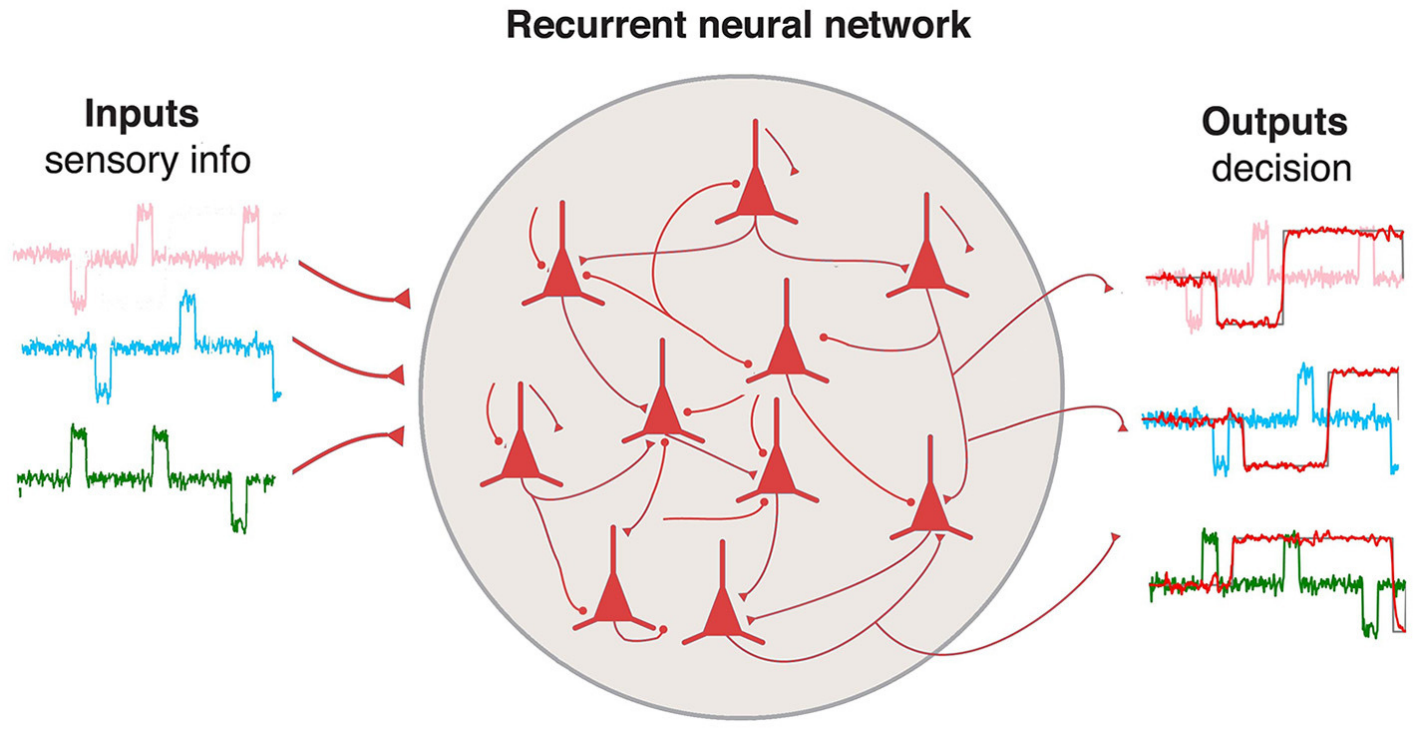
Schema for the RNN described by Equations 1 and 2. The network has three inputs and outputs to build the different memory states for the 3-bit Flip Flop task.

A simple schema of the model is presented in Figure 2. The network have three inputs and three outputs corresponding to the inputs and memory states of the 3-bit Flip Flop task.

As described in Section 1, the model is implemented in Python using Keras and Tensorflow (Abadi et al., 2015; Chollet et al., 2015). This allows us to use all current algorithms and optimization methods developed and maintained by a massive research community. Tensorflow has a recurrent layer directly implemented to represent Equation 7, where it is possible to choose the initialization of the parameters, number of units and activation function. This is shown in the following code box.

**Code for a Recurrent layer defined in Tensor Flow.**


tf.keras.layers.SimpleRNN(units,  activation="tanh",
    kernel_initializer="glorot_uniform",
    recurrent_initializer="orthogonal",**kwargs

The time scale of the Equation 7 is arbitrary. If we are interested, for example, in scales related to cognitive processes, we can consider 1 ms of temporal resolution.

The RNN can be initialized with different weight distributions. Several options can be selected using TensorFlow. This choice will depend, on the one hand, on the existence of some physical motivation or hypothesis of the models. On the other hand, from the ML perspective, it will depend on the performance associated with the considered initialization.

To build an RNN with the topology shown in Figure 2, it is possible to define a sequential model with layers such as:

**Code for the sequential model defined for the network in Figure 2.**


model = Sequential()
model.add(SimpleRNN(units=N, input_shape=(None,3),activation="tanh"))
model.add(Dense(units=3,input_dim=N))

Where input_shape = (None, 3) means the shape of the input vector, activation = 'tanh' corresponds to the definition of activation function, and Dense is a fully connected output layer. In this way, we completed the first step which is of the model definition in terms of the code. Orher acivation funcions are avaliable at https://www.tensorflow.org/api_docs/python/tf/keras/activations.

Other network architectures, such as Gated Recurrent Units (Chung et al. (2014)) or Long Short Term Memories (SHI et al., 2015), could be selected if there was any motivation from the perspective of the mechanisms to take into account. Both are already implemented in TensorFlow. Such code options are shown in the boxes below.

**Code using other architecture (GRU) for the sequential model defined for Figure 2.**


model = Sequential()
model.add(layers.GRU(units=N, return_sequences=True))
...

**Code using other architecture (LSTM) for the sequential model defined for Figure 2.**


model = Sequential()
model.add(layers.LSTM(units=N, input_shape=(None,3)))
...

The choice of the appropriate architecture will depend on the system to be modeled. Particular features, such as bias terms, can be also considered. In some cases, it is possible, to build architectures with additional features that are not pre-defined. This can be done by using the class structures in the framework.

For example, it is possible to define your own RNN cell layer (the inner part of the for loop) with custom behavior and use it with the generic keras.layers.RNN layer (the for loop itself). For more details see: https://www.tensorflow.org/guide/keras/working_with_rnns.

## 3 Task selection and parametrization

The parameterization of the task will have strong consequences on the possible dynamics obtained from the system through network training (Jarne and Laje, 2023). Possible examples are considering training with noise vs. without noise. Another is to consider amplitude variations or pulses of variable width in the training set.

Previous works have considered some relevant tasks in Computational Neuroscience related to decision-making or working memory. For example in Jarne (2021, 2022); Jarne and Laje (2023). All these processes use time-varying signals, which are very different from the binary boolean operations considered with forward networks. There are other examples of widespread tasks also considered in Computational Neuroscience, such as “Perceptual Decision Making” (Britten et al., 1992) or “Context-dependent Decision Making” (Mante et al., 2013). Each task has different possible parameterizations. In particular, the task defined in Mante et al. (2013) has recently been used to study the cortex response (Zhang et al., 2021).

It is also possible to consider working memory tasks such as “Delay match to sample with two items” (Freedman and Assad, 2006) or “Parametric working memory” (Roitman and Shadlen, 2002). For present work, motivated by Sussillo and Barak (2013), a working memory task, a 3-bit Flip Flop was chosen.

A Flip Flop is a binary task designed to store one bit of information. It has two inputs, Set (S) and Reset (R), which in our case will be represented by the temporal signal states +1 and -1 in amplitude, and one output Q. The output represents the current state of the Flip Flop and can be either 1 or -1. The operation is based on the following rule: If the input is 1, the Flip Flop output is set to the “1” state. If input is -1, the Flip Flop output is reset to the “-1” state. If both inputs are 0, the Flip Flop remains in its current state.

Once the task is chosen, the requirements must be translated into an algorithm that allows us to generate the training set. To parameterize the task, the following criteria were applied here:

- The possible states of the Flip Flop are represented in such a way that a positive pulse represents a set and a negative pulse represents a reset.
- The state of the output will change corresponding to the input command.
- A certain delay in the response was considered after the falling edge of the input signal.

The training data set consists of time series with pulses of fixed duration that represent set and reset signals. Those signals can be activated randomly and are separated by a random time interval. In all time series, a certain noise level has been added to the input. Each input elicitate a target output according to the Flip Flop rule: if we have a set signal or positive pulse, the output is in a high state. If we have a reset signal or negative pulse, output is in a low state. Otherwise, the output remains in the previous state.

The number of inputs in the network corresponds to the number of memory states that can be stored. A Flip Flop is a one-bit memory, meaning that two states only can be stored. In this way, we have registers formed by three Flip Flops (a 3-bit memory), which means that we have 8 different memory states.

**Figure 3:**
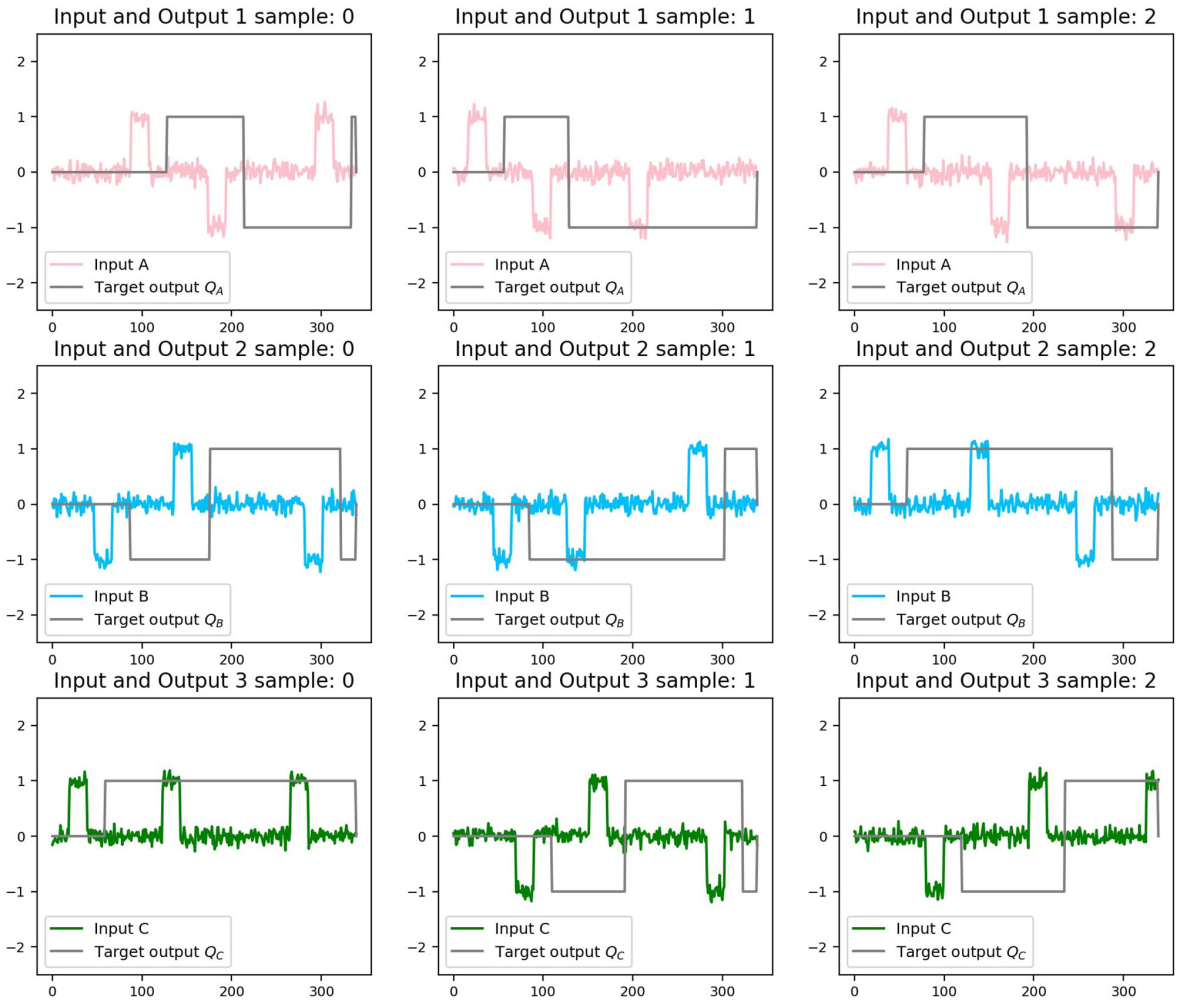
Three random samples of the training data set for each input channel. Each row (and color) corresponds to one of the inputs. Each column corresponds to a different sample. The gray line in each case represents the target output.

To complete the full training data set, it is necessary to generate tensors of size sample_size with the input time series of length time_series_lengh for each of the three inputs and outputs. To do that efficiently, we used Numpy arrays (Harris et al., 2020). In the present work, we provide the code to generate a Flip Flop data set. Three random components of the set x_train-y_train are shown in Figure 3. Input has amplitude noise of 10%. The target output, y_train, was simulated with a time delay answer of 20 ms. Each row (and color) corresponds to one of the inputs, and each column to a different sample. Each training sample consists of a Numpy array (Harris et al., 2020). This is shown in the following code box.

**Training data set pairs defined as Numpy arrays.**


x_train[sample_size,time_series_lengh,3]
y_train[sample_size,time_series_lengh,3]

## 4 Training protocol and parameter selection

Training methods for neural networks can be unsupervised or supervised. We focused on applying a supervised method.

Different approaches are available, but those in which a particular type of gradient descent method is applied stand out in the literature. An example is the paradigm of Reservoir Computing, specifically the use of liquid (or echo-state networks) (Maass et al., 2002), where the modifications of the network weights are made only in the weights of the output layer, *W*^*out*^.

Other outstanding approaches were developed by Sussillo and Abbot. They have developed a method called FORCE that allows the reproduction of complex output patterns, including human motion-captured data (Sussillo and Abbott (2009)). Modifications to the algorithm have also been applied successfully in various applications (DePasquale et al., 2018; Ingrosso and Abbott, 2019; Engelken et al., 2022).

The other method used for estimation of the gradient in RNNs is called Backpropagation Through Time (BPTT), and then an optimization method for minimizing the gradient. Given the recent advances in the implementation of this method with the open-source libraries previously mentioned, this is the method used here. Other back propagation-based methods have been published more recently. For example, in Khan et al. (2018), authors proposed to use fractional calculus to improve the conventional BPTT.

In this work, supervised learning was used, with standard backpropagation through time. An Adaptive Stochastic Gradient Descent training method provided by the Keras framework (Kingma and Ba, 2014) was applied.

First, recurrent weights were initialized using a random normal distribution with the orthogonal condition on the matrix. During training, noisy square pulse signals were used as the inputs, as the examples shown in Figure 3, and described in Section 3. In this way, sets of time series with 350 time points were generated containing random positive and negative pulses, with their corresponding output according to the operating rule described for the Flip Flop.

The appropriate loss function to train the model is the mean square error between the target function and the output of the network. It is defined as:


(8) $$\begin{array}{l} E\left(w\right)=\frac{1}{2}\overset{M}{\underset{t=1}{\sum }}\overset{L}{\underset{j=1}{\sum }}|{\text{Z}}_{\text{j}}\left(t\right)-{\text{Z}}_{\text{j}}^{\text{target}}\left(t\right){|}^{2}, \end{array}$$

where ${\text{Z}}_{\text{j}}^{\text{target}}\left(t\right)$ is the desired target function and **Z**_**j**_(*t*) is the actual output.

The training set consisted of more than 15000 different random samples. The previously mentioned training procedures correspond, in terms of the code structure, to the methods for compiling and fitting models. The loss function and the optimizer algorithm are chosen in the compiling step. Different information about the training data set, epochs, and other training characteristics can be specified with the fitting method. An example of implementation is shown in the following code box.

**Code for the compiling and training steps.**

 
model.compile(loss = "mse", optimizer=ADAM)
model.fit(x_train[50:sample_size,:,:], y_train[50:sample_size,:,:],
 epochs=epochs, batch_size=128, shuffle=True)
 

The main parameters of a neural network are the weights of the connections. These parameters are learned during the training stage. On the other hand, hyperparameters are parameters of your neural network that can not be learned via gradient descent or some other training method. These include the learning rate, number of layers, or the number of neurons in a given layer.

Tuning the hyperparameters means the process of choosing the best values of them. Typically, this is done by evaluating the performance of the network on a validation set. Then, we have to change the hyperparameters and re-evaluate the model, choosing the values that give the best performance on the validation set. Another approach for choosing them is to have an informed decision or hypothesis related to the physics or nature of the system under study.

How do we choose these values? Often, there is good standard initialization related to each particular task of interest. An example of the criteria is provided for the Flip Flop task in Table 1.

**Table 1:** Model's parameters and criteria for the network's implementation and training.

**Parameter/criteria**	**Value**
Units	400
Time step	1
Input Weight	3 × 400
Recurrent Weights	400 × 400
Output Weight	400 × 3
Training algorithm	BPTT ADAM
Initialization	Random Orthogonal
Regularization	None

Another aspect to consider is the regularization of the model. Regularization refers to training our model well enough that it can generalize over data it hasn't seen before.

To summarize, in the training stage, the main aspects we have to consider are the size of the network, data set, noise, and regularization terms that are appropriate for the considered task.

A good practice is to build a set of RNNs (at least a dozen) with different hyperparameters that are correctly trained to perform the same task and that can serve as a test set and allow us to compare the variations in the possible solutions.

## 5 Analyzing the results

The results obtained after training the RNN can be analyzed in several ways. On the one hand, we can consider the quality of the solutions obtained (and the robustness) by analyzing the predicted output concerning the target and stability against noise conditions. On the other hand, we can study the solutions in terms of the dynamics and collective behavior. In Section 5.1, we will first briefly discuss how to evaluate robustness in terms of the output obtained and how robust it can be against variations in the input stimuli. Then, in Section 5.2, we will discuss in-depth details of the dynamics and collective behavior.

### 5.1 Evaluating RNN performance and robustness

We can measure the rate of success for a set of networks in terms of Euclidean distance between target and output (Jarne and Laje, 2023). The distance between the network's predicted output and the target output could be estimated using the Numpy function linalg.norm(), which in this case is the Frobenius norm (or Euclidean norm) between the output vector of the trained network and the target output. We could use other metrics, such as Mean squared error (MSE), which measures the average squared difference between predicted and actual values.

**How to use linear algebra library from Numpy to calculate the norm of the vector “Difference” using the Euclidean norm.**


from numpy import linalg as LA
euclidean_norm = LA.norm(Difference)

We can also include noise and variations in amplitude to the data and find constraints on how the network is still able to accurately predict the target.

In addition to the metrics mentioned above, we can also evaluate the robustness using other available Accuracy metrics.

In general, what type of data sets we use to train the network is directly related to how robust we want to design our systems and what properties we are seeking to represent and should be taken into account when comparing the abstract models studied here with those obtained from experimental data.

Characterizing the limitations of the network concerning variations in input signals is a good practice that will allow us to better understand the results obtained.

Another analysis related to robustness that can be performed is to determine the minimum size of the network that allows the parameterized task to be performed (in our example Flip Flop), given a certain desired accuracy.

### 5.2 Analyzing the collective behavior of trained RNNs: visualization and dimensionality reduction techniques

After training, we obtained a set of RNNs that can perform the tasks of interest. We describe in this section the different aspects to analyze regarding the network's collective behavior. We selected a method for the model's visualization and a group of tools to extract the relevant information.

**Figure 4:**
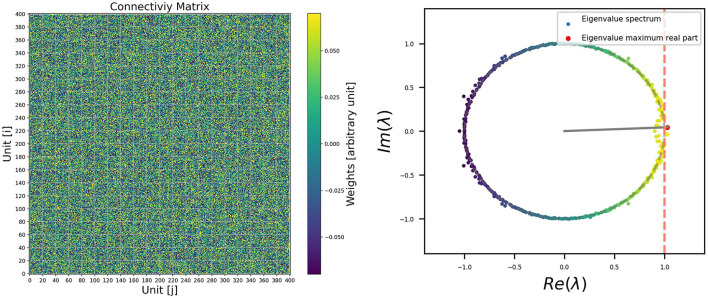
**Left**: Example of visualization for the connectivity matrix for a trained RNN. **Right**: Eigenvalue distribution of ***W*^*rec*^** in the complex plane.

For example, it is possible to visualize the connectivity matrix (recurrent weight matrix), as it is shown on the left side of Figure 4. The columns represent the output connection of the i-neuron, and the rows are the input connection. They are also called post-synaptic and pre-synaptic. The color bar on the right side represents the intensity of the connections. We have to consider an appropriate scale for the visualization. Even so, it may not be entirely clear how to observe the relevant information, apart from the fact that, after training, most of the weights remain close to zero. As a first approach for visualization, a plot of the connectivity matrix could be useful, even if the case presented here does not reveal relevant information. It's important not to undervalue it. If the connectivity has some structure farther from a random distribution, it will be observed in the connectivity plot. For example, having null autoconnection terms will be reflected in the color of the diagonal terms of the matrix plot. Another example could be sparsity, which would be reflected in patches over the matrix. Or perhaps, in the case of having excitatory and inhibitory units, it would be easy to visualize the different columns corresponding to the same sign of out connection. In case of imposing such constraints on the connections, as Dales' Law (Dale, 1935), or any particular constraint, they will be visible in this stage, and this representation will be more useful.

If we don‘t obtain relevant information with this first visualization, we know that useful information could be still encoded in the connectivity matrix, but it may not be immediately distinguishable with a connectivity plot. There are different transformations or analyses that we can perform on the recurrent weight matrix with this aim. Different Linear Algebra operations are available in the Numpy Library (Harris et al., 2020) that are optimized to be used with the array structures. For example, if we perform a decomposition of *W*^*rec*^ in their eigenvectors and eigenvalues, we can obtain the eigenvalue distribution as it is shown in the right side of Figure 4. This analysis can be done using the code in the following code box.

**How to use linear algebra library from Numpy for eigenvalue decomposition.**


from numpy import linalg as LA
eigenvalues, eigenvectors= LA.eig(Matrix)

During training, the matrix associated with the network tends to be non-normal, which results in their eigenvalues lying closer to the unit circle. This behavior is explained in more detail in papers that study the dynamics of RNNs, where it is shown that the presence of recurrent connections and the attractors in the network's dynamics can cause this accumulation of eigenvalues close to the unit circle (Asllani et al., 2018; Bondanelli and Ostojic, 2020; Jarne, 2022).

Additionally, in these studies, it is typically shown that this accumulation of eigenvalues on the unit circle leads to slowing down the dynamics of the network. They can be linked to the emergence of long-term memories related to the linearization of the system. Therefore, this behavior can be understood as a necessary condition for the network to effectively store and retrieve information over longer time scales.

**Figure 5:**
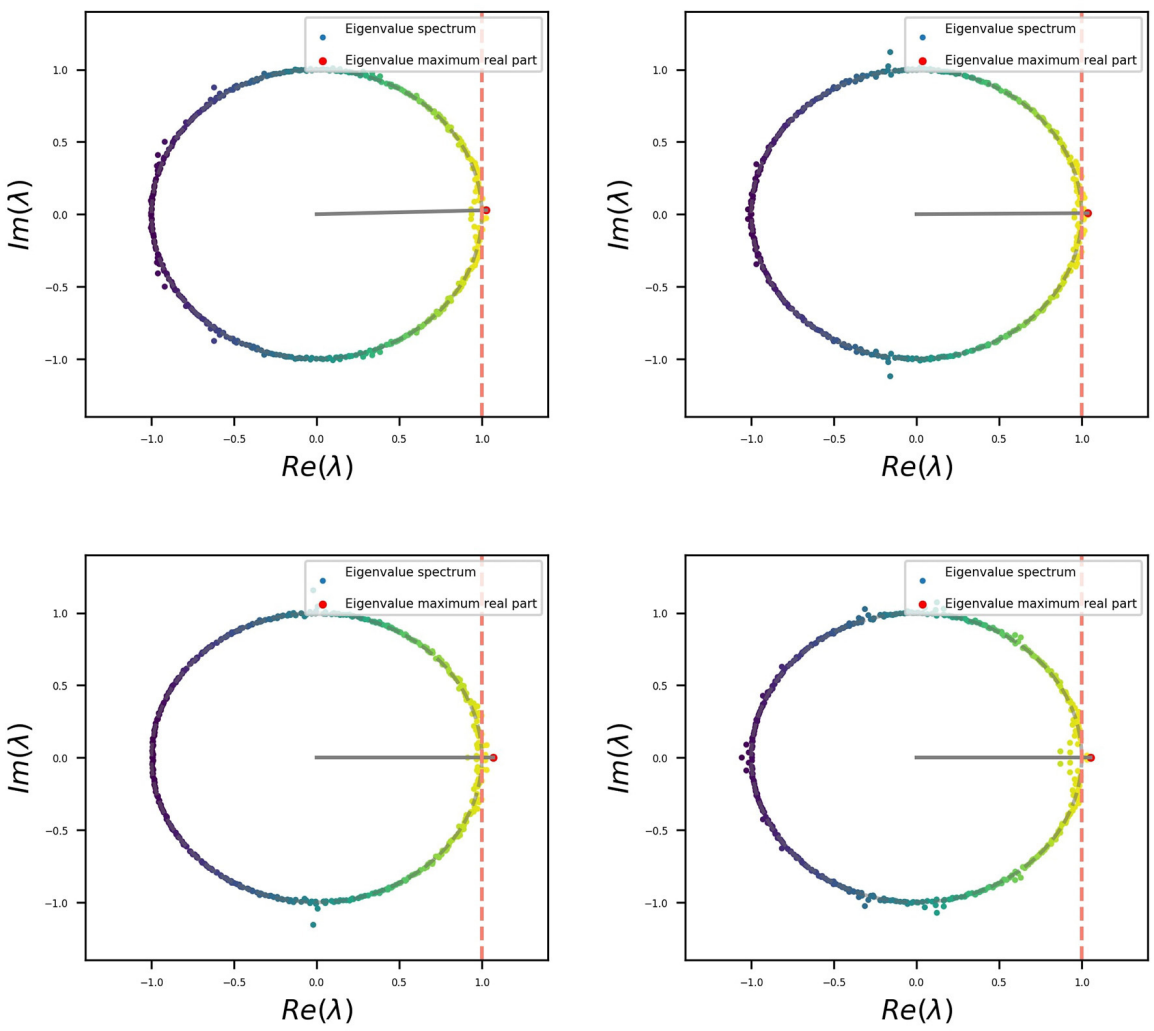
Four different examples of eigenvalue distributions of *W*^*rec*^ for trained RNNs which have been initialized before with an orthogonal distribution.

In the case presented here, we can visualize that, except for a small group of eigenvalues that migrated out of the unit circle, the rest remain on it, which is related to the initial orthogonal condition. The same was replicated throughout all simulations. A set of four examples is shown in Figure 5, and the code provided allows us to reproduce more. Eigenvalues outside the unitary circle seem to be related to the behavior (or modes) observed for the different stimuli at the input as described in (Jarne, 2022). This is relevant in terms of the dynamics. Additional information related to the connectivity matrix could also be obtained (Jarne, 2022).

Other possible studies that we can perform are related to the response in terms of the activity of the network units when applying the different stimuli.

Since we have a large number of units, and for each, an activity vector, dimensionally reduction methods are appropriate to analyze such behavior. They have been used widely in different works related to large-scale neural recordings (Cunningham and Yu, 2014; Williams et al., 2018).

Scikit-learn (Pedregosa et al., 2011) is a Python open-source library based on Numpy that allows us to perform dimensionality reduction, feature extraction, and normalization, among others. It has efficient methods for predictive data analysis. A possible decomposition could be, for example, Principal Component Analysis (PCA) or also Single Value Decomposition (SVD). The following code box shows how to call the library's functions.

**How to import scikit learn libraries to perform single value decomposition and principal component analysis.**


from sklearn.decomposition import PCA
from sklearn.decomposition import TruncatedSVD

These tools can be used to extract relevant features of the system. For this work, the behavior, in terms of the activity of the units, was analyzed.

It is well known that the different memory states in a 3-bit memory are distributed in the vertex of a cube-like form in the space state (Sussillo and Barak, 2013). This was shown when authors explored the hypothesis that fixed points, both stable and unstable, and the linearized dynamics around them, can reveal aspects of how RNNs implement their computations.

**Figure 6:**
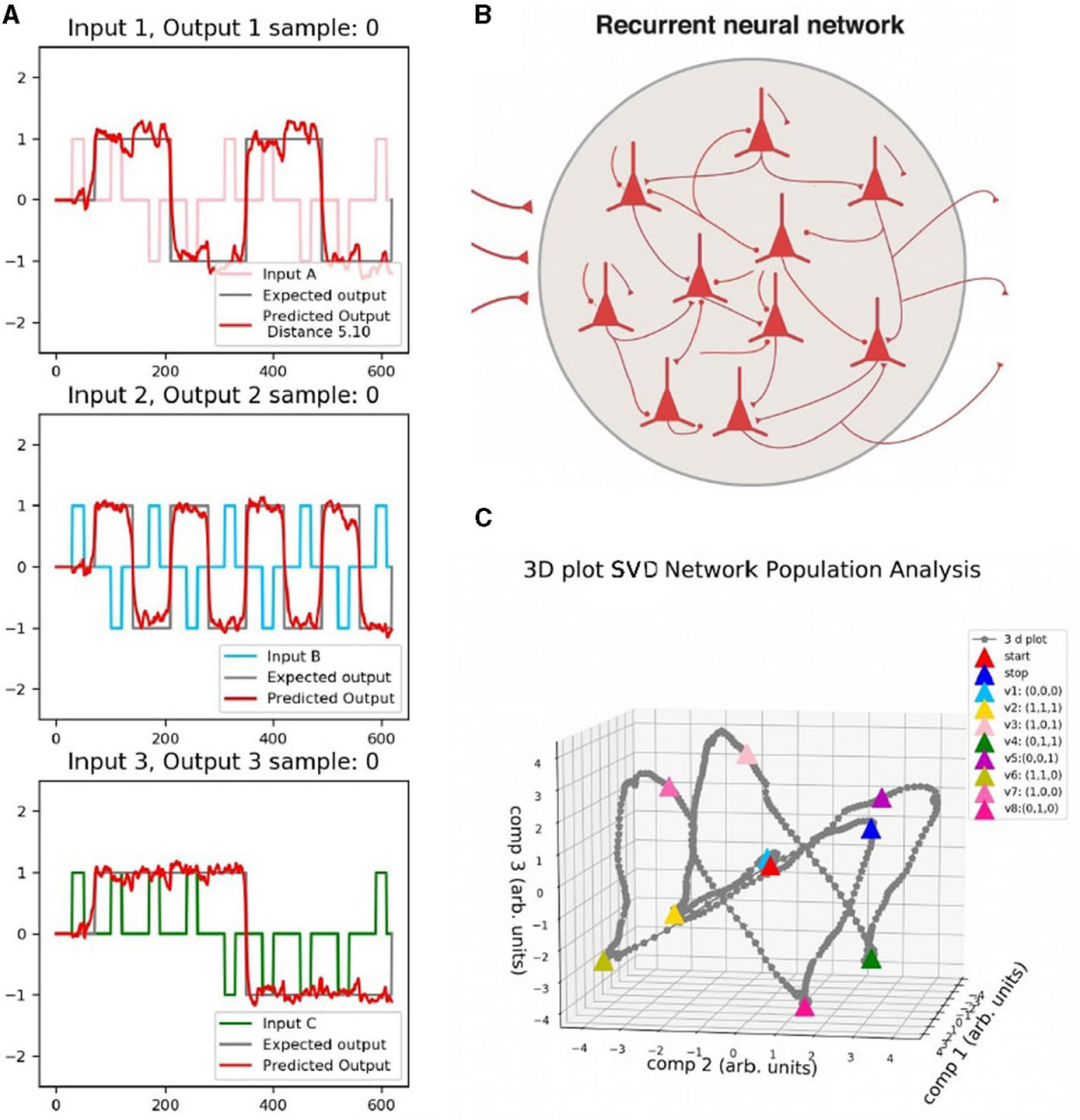
**(A)** Data set for testing. Each panel corresponds to one input and the predicted output of the Flip Flop, which is shown in red. **(B)** Schema of the network. **(C)** Single Value Decomposition applied to the activity vector **H**(*t*) of panel b) in the three components of greatest variance. Each color point corresponds to a different memory state.

A data set was built for testing and reproducing the behavior. It generates eight different memory states, as shown on the left side of Figure 6, where each panel shows the input and output of the network. Time series of 600 ms were considered to generate all the different memory states of the 3-bit memory by choosing the correct commutation for the inputs in fixed time intervals. Output responses are shown in red in the figure.

The testing set is injected into the network (right upper panel of the figure), and then the activity of the units is analyzed by applying SVD on the activity vector *H*(*t*). The behavior of the system was represented in the three axes of the greatest variance. The bottom right part of Figure 6 shows the activity in the reduced state space (3-dimensional). Each vertex corresponds to each memory state marked in different colors.

**Figure 7:**
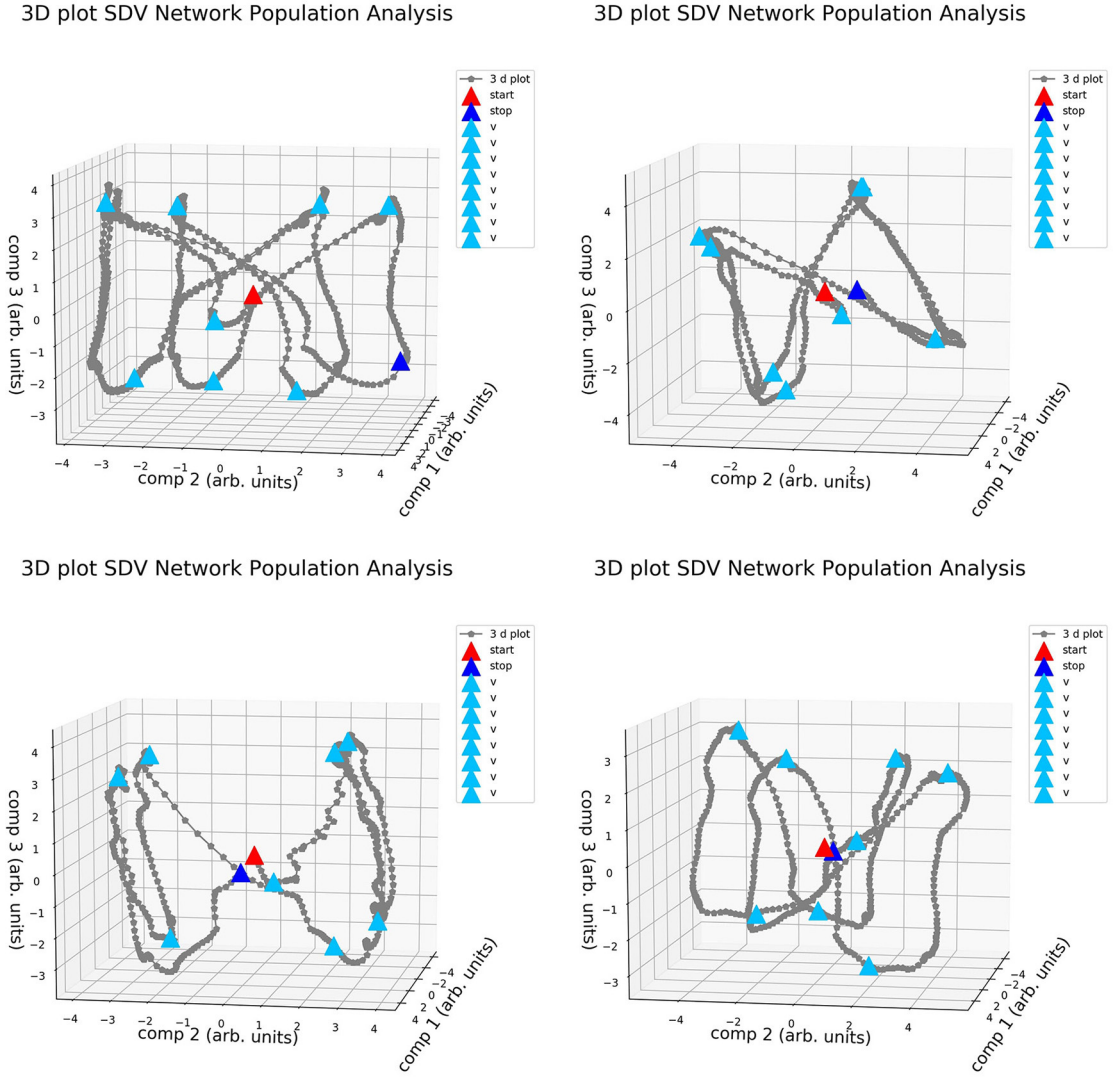
Activity of the four different realizations of RNNs trained for the same 3-bit Flip Flop task in the dimensionally reduced space. Configurations are similar to one presented in Figure 5.

It is well known that different variations of the realizations, in terms of weight distribution and dynamical behavior, are possible when training networks for the same task (Jarne, 2021, 2022; Jarne and Laje, 2023). This was exemplified in Figure 5 and is also shown in Figure 7, where the four different realizations of the trained networks of Figure 5 were elicited with the same testing data set and a decomposition SVD analysis, was performed.

The vertices in this space of the main components are distributed in different positions. A cube-like structure always appears, similarly to what was observed in Sussillo and Barak (2013), and is rotated in different spatial directions for different realizations. It is possible to study and classify the behavior of the obtained systems by comparing the network obtained. This cube-like structure is characteristic of this task parametrization, and it appears even when we used a different training method and network parameters compared with previous studies (Sussillo and Barak, 2013).

Additional analysis could be considered depending on the aspects of interest to be studied. Here, a minimal analysis was proposed. We described in detail the steps, visualization tool, criteria, and implementation. The code for training and analysis is provided also in an open repository: https://github.com/katejarne/3-bit-FF-tutorial/tree/main/paper. It can be also used as an open framework to parametrize different tasks or additional studies. In this way, we can generate and compare the different realizations for the Flip Flop task or define different tasks for study.

## 6 Conclusions

In this work, all steps to build and analyze an RNN have been presented for a sample task. We started from the model description in terms of the equations, discretization, and code implementation. We discussed different options that are available for code implementation depending on the considered model and scientific questions. Then, we described the task parametrization and network training protocol. We also presented a set of tools to analyze the results using open-source scientific libraries making use of the different visualization tools that allow extracting relevant features.

We used the Flip Flop task as an example, but other relevant tasks could be considered, as mentioned in Section 3. For example, “Perceptual Decision Making” (Britten et al., 1992), “Context-dependent Decision Making” (Mante et al., 2013; Zhang et al., 2021), working memory tasks such as “Delay match to sample with two items” (Freedman and Assad, 2006) or “Parametric working memory” (Roitman and Shadlen, 2002). In this work, motivated by Sussillo and Barak (2013), a working memory task such as a 3-bit Flip Flop, was chosen to show the entire process: from the differential equations of the RNN model, discretization, through the parameterization of the task and the methods of analysis for the activity of the network against the different stimuli on the network.

The use of open-source scientific frameworks designed and maintained for large communities, such as the tools used here, allows enhancing research. This is why we are currently using tools that are more transparent in terms of code and documentation because they are open to being modified and improved by thousands of users.

Regarding the limitations, the proposed method was evaluated on a single cognitive task, namely Flip Flop. It is not clear whether the proposed pipeline would generalize to other more complex types of cognitive tasks. We did not include other explicit biological constraints in this example. We could extend it to include sparsity or Dale's law, for example. Further work could address such research directions to complement generalization and biological details.

## Data availability statement

The datasets presented in this study can be found and also created using the online repository: https://github.com/katejarne/3-bit-FF-tutorial.

## Author contributions

CJ: Conceptualization, Formal analysis, Investigation, Methodology, Resources, Software, Validation, Visualization, Writing – original draft, Writing – review & editing.
